# Medical genetics and genomic medicine in Nigeria

**DOI:** 10.1002/mgg3.419

**Published:** 2018-06-05

**Authors:** Adebowale A. Adeyemo, Olukemi K. Amodu, Ekanem E. Ekure, Olayemi O. Omotade

**Affiliations:** ^1^ National Human Genome Research Institute National Institutes of Health Bethesda Maryland; ^2^ Institute of Child Health College of Medicine University of Ibadan Ibadan Nigeria; ^3^ Department of Paediatrics College of Medicine University of Lagos Lagos Nigeria

**Keywords:** Africa, genetics, genetic testing, genomics, medical genetics, Nigeria

## Abstract

Medical genetics and genomic medicine in Nigeria.

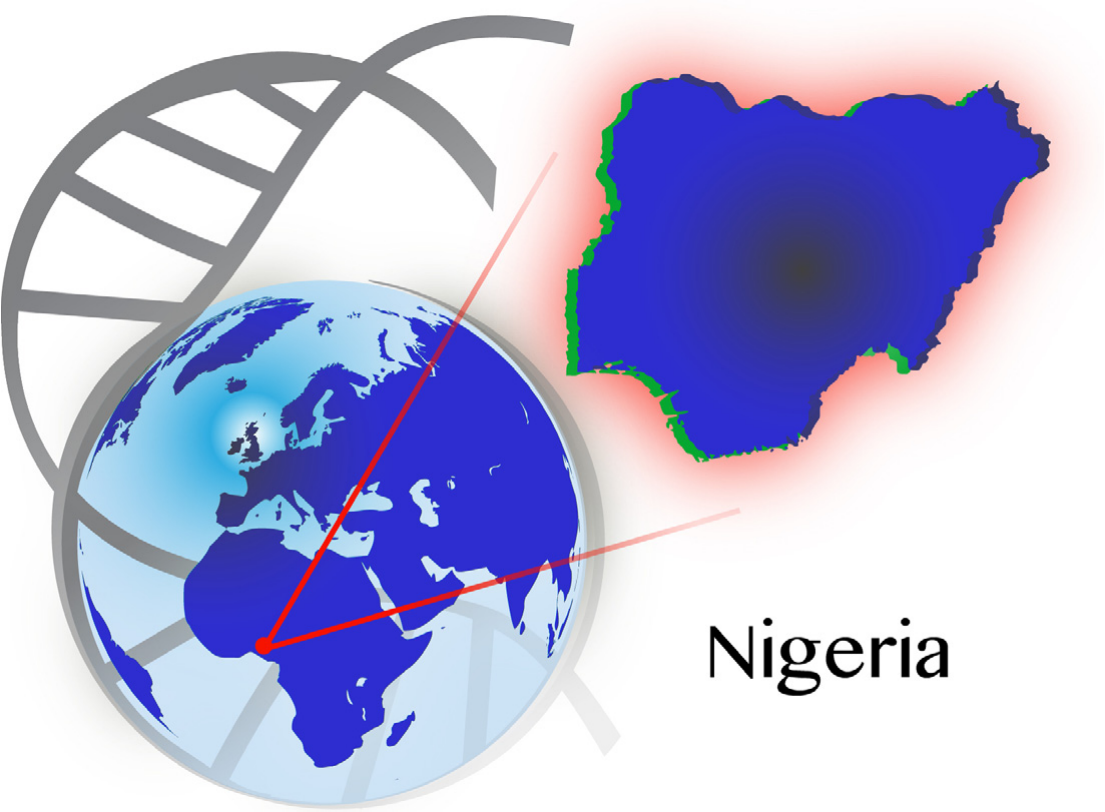

## 1 INTRODUCTION

Nigeria is located in West Africa and shares land borders with the Republic of Benin in the west, Chad and Cameroon in the east, and Niger in the north. It has a land area of 910,768 square kilometers and a water area of 13,000 square kilometers. Archeological research indicates that the geographical area that is currently Nigeria was inhabited as early as 11,000 BC and probably earlier (Harvati et al., 2011; Shaw & Daniells, 1984). The earliest identified Nigerian culture is the Nok culture that thrived between 1500 BC and 200 AD on the Jos Plateau in northeastern Nigeria. Arab traders arrived in Northern Nigeria by the 9th century AD, while the first Europeans were Portuguese explorers who arrived in southern Nigeria in the 1470s. Nigeria was a British colony from the early 1900s until she gained independence in 1960. Administratively, the country comprises of 36 states and the Federal Capital Territory, Abuja. The climate varies ranging from equatorial in the south to tropical in the center and arid in the north. Nigeria has the largest economy in Africa with its source of foreign exchange and government earnings coming largely from the oil sector. The country is classified as a lower middle income country and it has an estimated GDP per capita of $5,900 (Indexmundi, 2018).

**Figure 1:**
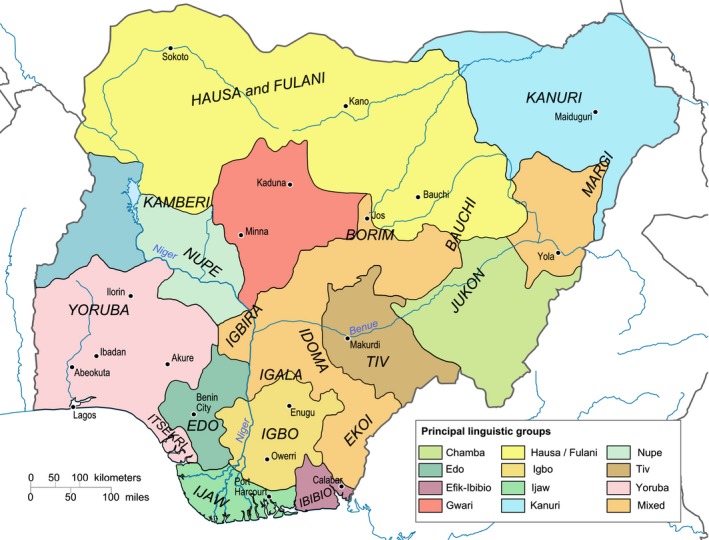
Ethno‐linguistic map of Nigeria. Source: Wikimedia Commons. Image by Hel‐hama [CC BY‐SA 3.0 ( https://creativecommons.org/licenses/by-sa/3.0)], https://commons.wikimedia.org/wiki/File:Nigeria_linguistical_map_1979.svg

Nigeria is the most populous country in Africa and the seventh most populous country in the world with an estimated population of 182 million people and an annual population growth rate of 3.5% (National Population Commission 2018). Due to the high fertility rate, the population is quite young and more than half of the population is <30 years of age. Estimated mortality rates are high (maternal mortality 814 deaths/100,000 live births in 2015; under‐five mortality 104.3/1,000 live births in 2016) and life expectancy is 56 years for females and 53 years for males (World Health Organisation 2018). About half of the population lives in rural areas but there is rapid growth of rural‐urban migration precipitated by various factors, including better employment, education, and business opportunities in urban areas. Nigeria is composed of more than 250 ethno‐linguistic groups, the most populous being the Hausa‐Fulani 29%, Yoruba 21%, Igbo (Ibo) 18%, Ijaw 10%, Kanuri 4%, Ibibio 3.5%, and Tiv 2.5% (Figure 1). The country has languages from three of the four major African language families: Afroasiatic, Nilo‐Saharan and Niger‐Congo (Ade‐Ajayi et al., 2018). The two major religions practiced in Nigeria are Christianity and Islam.

## 2 THE HEALTH SECTOR, NATIONAL HEALTH POLICY, AND GENETICS SERVICES

Nigeria's health sector consists of a mix of public (government) and private facilities. There are three strata of health care: primary, secondary, and tertiary with the last (university teaching hospitals and specialist hospitals) comprising the highest level of health care. Total expenditure on health as a percentage of GDP in Nigeria was 3.7% in 2014. There is a national health insurance scheme that was established by decree in 1999 and was later re‐enacted as the National Health Insurance Scheme Act Cap, N42 Laws of the Federation of Nigeria 2004. The scheme was launched in 2005 with the aim of providing good‐quality health care to Nigerians at an affordable cost through various prepayment systems. However, participation is not mandatory and it is estimated that the scheme currently covers a small fraction of the population. Therefore, health insurance coverage is not yet universal and Nigerians have to pay out of pocket for most health care expenses. Nigeria's National Health Act 2014 was signed into law on October 31, 2014 (Enabulele & Enabulele, 2016). It provides a legal framework for the regulation, development, and management of Nigeria's Health System and sets standards for rendering health services in Nigeria and for related matters. Most national health policy documents do not directly address genetic services or genomic medicine, with the exception of sickle cell disease as described below.

## 3 COMMON GENETIC DISORDERS IN NIGERIA

Sickle cell disease (SCD) is probably the single most common severe genetic disorder in Nigeria, occurring in up to 2%–3% of newborns. Sickle cell trait affects up to a quarter of the population and surveys starting from the 1950s show a stable prevalence of 22%–25% for the trait (Akinyanju, 1989; Fleming et al., 1979; Jelliffe & Humphreys, 1952; Kaine & Udeozo, 1981; Nwogoh, Adewoyin, Iheanacho, & Bazuaye, 2012; Omotade et al., 1998). Given Nigeria's population of ~180 million, Nigeria probably has the largest population anywhere of people affected by SCD or at risk of having children with SCD. Another common monogenic disorder is glucose‐6‐phosphate dehydrogenase (G6PD) deficiency. The form of G6PD deficiency commonly seen in Nigeria is the African type (GdA‐) and it manifests primarily as increased risk of neonatal jaundice and drug‐induced hemolysis. Important contributions to knowledge about G6PD deficiency were made as a result of research done in Ibadan (Luzzato & Allan, 1968; Bienzle, Sodeinde, Effiong, & Luzzatto, 1975; Luzzatto, Sodeinde, & Martini, 1983; Sodeinde, 1992; Sodeinde, Chan, Maxwell, Familusi, & Hendrickse, 1995), which was also the site of the first neonatal screening program for G6PD deficiency by electrophoresis. Oculocutaneous albinism is reportedly common in the eastern part of the country (Hong, Zeeb, & Repacholi, 2006; King, Creel, Cervenka, Okoro, & Witkop, 1980; Okoro, 1975). However, there are currently no comprehensive molecular genetic studies that estimate its prevalence and distribution. Congenital malformations are quite common and they account for substantial morbidity and mortality in childhood (Adeyemo, Okolo, & Omotade, 1994; Adeyemo, Gbadegesin, & Omotade, 1997; Ekanem, Bassey, Mesembe, Eluwa, & Ekong, 2011; Obu et al. 2012, Singh, Chukwunyere, Omembelede, & Onankpa, 2015; Ekwochi et al., 2017). Autosomal trisomies, mainly trisomy 21 (Down syndrome), are also seen frequently (Adeyokunnu, 1982a,b, 1983). Overall, a comprehensive picture of the relative burden of various genetic disorders in Nigeria is lacking given the absence of newborn screening, the paucity of diagnostic facilities (Ahmed, 2004) and lack of population‐based registries.

## 4 MEDICAL GENETICS IN NIGERIA

The medical literature from Nigeria shows case reports or case series of genetic disorders from clinical observations. For example, a case of dystrophia myotonica in a Nigerian family was reported in 1973 (Dada, 1973) and the first case of hereditary anhidrotic ectodermal dysplasia in Africa was reported in a Nigerian family (Familusi, Jaiyesimi, Ojo, & Attah, 1975). However, the first medical genetics service in Nigeria was established in the early 1970s in Ibadan with a clinical and cytogenetics service. This unit, staffed by a pediatric clinical geneticist and a cytogeneticist, provided clinical genetics services and conducted seminal research on the autosomal trisomies, skeletal dysplasias, and other dysmorphic disorders (Adeyokunnu, 1981, 1982a,b,c, 1983; Adeyokunnu & Adeniyi, 1981; Adeyokunnu et al., 1975). The work done by this unit also laid the foundation for subsequent genetics services and research in Ibadan.

**Figure 2:**
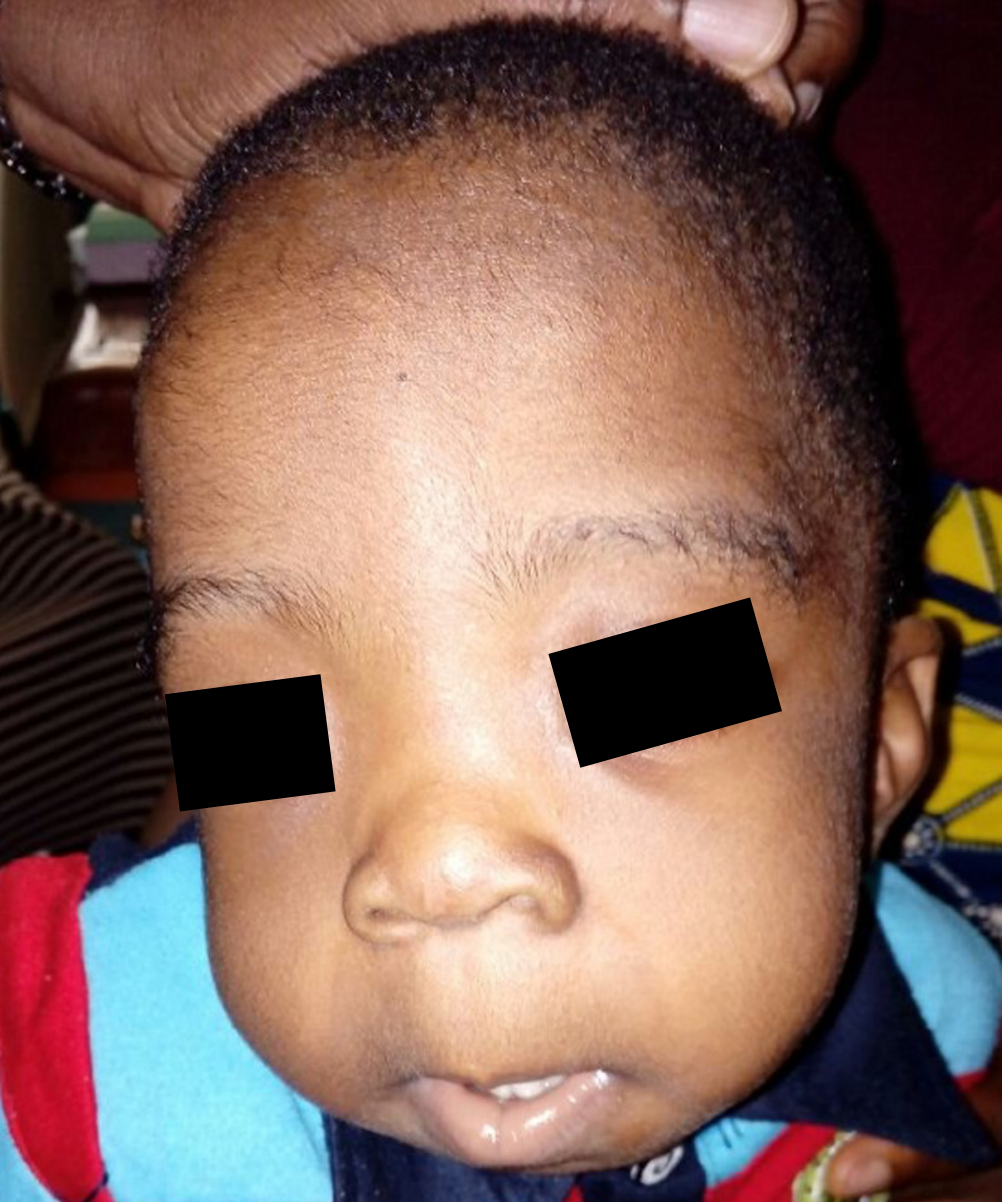
A Nigerian child with Hajdu–Cheney syndrome. The syndrome is a rare autosomal dominant disorder of connective tissue with clinical features that include joint laxity, bone deformities, short stature, small maxilla, hypoplastic frontal sinuses, delayed acquisition of speech and motor skills, and dolichocephalic skull

Currently, there are few medical geneticists and genetic counselors in Nigeria. Most patients with genetic disorders are seen by pediatricians (some with an interest in genetics), while others are seen by specialists (Figure 2). Facilities for molecular diagnosis (including prenatal diagnosis) also remain suboptimal and are not widely available. All these factors mean that opportunities for training in medical genetics are not available within the country and prospective trainees have to travel outside the country. There are several centers (both public and private) that can perform DNA extraction. However, few laboratories are able to do routine genotyping or sequencing for patients on‐site and most molecular diagnosis still has to be done outside the country. A few laboratories, notably that of the Sickle Cell Foundation of Nigeria can do PCR‐RFLP analysis for the sickle cell mutation.

## 5 THE ROLE OF SCD

SCD occupies a unique role in medical genetics in Nigeria due to its high prevalence and its devastating effect on morbidity and mortality. Therefore, it has rightly received more attention than any other genetic disorder. For example, most medium‐to‐large health facilities run a dedicated sickle cell anemia clinic. Also, several hospitals conduct infant screening for SCD by hemoglobin electrophoresis at 9 months (i.e., see Omotade et al., 1998). The high prevalence of the disorder means that every clinician trained in the country is familiar with its genetics, clinical presentation, and management. Awareness of SCD is quite high in the general population, in whom premarital testing and counseling is becoming increasingly common. Indeed, some churches require hemoglobin type results before agreeing to conduct weddings. SCD is also the only genetic disorder for which there are federal government guidelines for its control and management (Federal Ministry of Health 2014). The guidelines included newborn screening for SCD using high performance liquid chromatography (HPLC), management of diagnosed cases, genetic counseling, and establishment of six comprehensive sickle cell centers in the country. However, it is uncertain how widely or how closely the guidelines are followed across the country. SCD in Nigeria is also unique in that it is one inherited disorder for which the need for prenatal diagnosis is recognized (Federal Ministry of Health, 2014), prenatal diagnosis is available in a few centers (Adewole et al., 1999; Akinyanju et al., 1999; Oloyede, Olaide, & Onyinye, 2014), and attitudes toward prenatal diagnosis for SCD have been studied (Adeola Animasahun, Nwodo, & Njokanma, 2012; Akinyanju et al., 1999; Durosinmi et al., 1997).

## 6 GENETICS RESEARCH IN NIGERIA

Genetics research in Nigeria has evolved over the decades. Early studies were mainly case series. Adeyokunnu (1982a) in a prospective study spanning 9 years reported an incidence of 1 in 865 livebirths of Down syndrome in Ibadan confirming that Down's syndrome is not a rare occurrence in Nigeria. Cytogenetic analysis of 386 cases showed that about 96% were regular trisomy 21, 2.5% were due to translocations, and 1.5% were mosaics. Similar studies established incidence at birth for trisomy 18, trisomy 13, and Turner syndrome (Adeyokunnu, 1982b, 1983). A retrospective study of neurodegenerative disorders including 2.1 million patients seen at the University College Hospital, Ibadan, Nigeria over a period of 25 years reported cases of hereditary ataxia, Huntington's chorea, ataxia telangiectasia, and Charcot–Marie–Tooth disease (Aiyesimoju, Osuntokun, Bademosi, & Adeuja, 1984). Adeyemo et al. (1994, 1997) reported the distribution of congenital malformations in children and their contribution to morbidity, including reporting a prevalence of 11.1% of congenital malformations in neonates presenting at a major university teaching hospital with the most common being spina bifida (22.5%), anorectal malformation (13.4%), omphalocele (9.9%), and tracheoesophageal fistula (8.5%).

Studies of molecular genetics markers in complex disorders such as hypertension, obesity, and diabetes began in the 1990s. For example, Rotimi et al. (1994) investigated the frequency of the 235T and 174M alleles of the angiotensinogen (*AGT*, MIM 106150) gene in Nigeria and also reported a significant association between the 235T variant and plasma level of the angiotensinogen protein and hypertension. Anderson et al. (1997) investigated apolipoprotein B insertion/deletion and XbaI polymorphisms in relation to variation in serum lipoprotein and lipid levels in two Nigerian populations. Subsequently, genome‐wide linkage studies were conducted in or included Nigerian populations, largely facilitated by investigator‐driven studies and international collaborations such as the International Collaborative Study of Hypertension in Blacks (ICSHIB) (Kaufman, Durazo‐Arvizu, Rotimi, McGee, & Cooper, 1996; McKenzie et al., 2005; Rotimi et al., 1995, 2006, 1997) and the Africa America Diabetes Mellitus (AADM) Study (Rotimi et al., 2001; Rotimi et al., 2004). These studies described linkage scans for obesity, diabetes, hypertension, and serum lipids, among others (Adeyemo et al. 2003, Adeyemo, Johnson, et al., 2005, Adeyemo, Luke et al. 2005; Chen et al., 2005; Rotimi et al., 2004; Rotimi et al. 2006, Hicks et al., 2007). Indeed, the AADM Study contributed to the discovery of the first robustly replicated locus for type 2 diabetes: *TCF7L2* (MIM 602228; Helgason et al., 2007). Nigerian populations were also included in subsequent studies such as genome‐wide association studies (GWAS) or post‐GWAS studies for various cardiometabolic disorders (Adeyemo et al., 2015; Chen et al., 2017; Kang et al., 2010; Nandakumar et al., 2017; Reder et al., 2012; Tayo, Luke, Zhu, Adeyemo, & Cooper, 2009; Tayo et al., 2013). Studies of host genetic risk factors for infectious disease have also been done in Nigerian populations, especially in relation to malaria (Amodu et al., 2005, 2012; Olaniyan et al., 2014, 2016), including some in the context of international consortia such as MalariaGen (Olaniyan et al., 2014; Clarke et al., 2017).

Recent studies that have focused more directly on medical genetic disorders include studies of orofacial clefts (cleft lip and/or palate), congenital deafness, and congenital heart defects (CHD). The first genetic study on nonsyndromic clefts in sub‐Saharan Africa was conducted in a Nigerian population (Butali et al., 2011). Subsequent studies from the same research group investigated rare variants in African populations by sequencing GWAS‐identified loci for orofacial clefts (Butali et al., 2013, 2014a,b; Eshete et al., 2018; Gowans et al., 2018). The investigators also reported novel IRF6 mutations in families with Van Der Woude syndrome and popliteal pterygium syndrome from sub‐Saharan Africa (Butali et al., 2014a,b). The first GWAS for orofacial clefts in Africa is currently under way. Molecular genetic studies of congenital deafness in Nigeria revealed that mutations in the known deafness genes [*GJB2* (MIM 121011)*, GJB6* (MIM 604418)*, SLC26A4* (MIM 605646)] that are common in Europe and America are uncommon among Nigerian deaf patients (Lasisi, Bademci, Foster, Blanton, & Tekin, 2014). Subsequent studies identified a novel mutation responsible for X‐linked deafness—c.987T>C (p.Ile308Thr) in *POU3F4* (MIM 300039), the first novel deafness gene among Nigerians (Bademci et al., 2015). An ongoing collaborative project is studying the genomics of CHD in Lagos using an approach that includes chromosomal array screening, whole exome capture and sequencing (WES) as well as functional studies. The distribution of CHD seen among the first 206 probands include: VSD (24.8%), atrial septal defects (8.2%) and Tetralogy of Fallot (21.3%). While the study is still ongoing, interim analyses show structural variation in one‐in‐seven patients (with the most common condition 22q11 deletion syndrome) and several candidate pathogenic mutations in genes known to cause CHD in animal models. While this is not an exhaustive list of such studies, it points to the increasing feasibility of conducting such studies in Nigeria. It should also be noted that Nigerian clinicians are participating in the NHGRI Atlas of Human Malformation Syndromes in Diverse Populations, an electronic dysmorphology atlas that aims to represent the diversity of features in patients with specific dysmorphologic syndromes seen around the world (Koretzky et al., 2016; Muenke, Adeyemo, & Kruszka, 2016). The atlas is facilitating comparative research for selected syndromes—including Down, Noonan, and 22q11.2 deletion syndromes (Kruszka, Addissie, et al., 2017, Kruszka, Porras, Addissie, et al. 2017, Kruszka, Porras, Sobering, et al. 2017)—and would be of immense benefit to the development of medical genetics in Nigeria.

## 7 NIGERIA IN INTERNATIONAL GENETICS AND GENOMICS INITIATIVES

Nigerian populations have been included in several international genomics projects that followed the sequencing of the human genome. For the International HapMap Project, the Yoruba ethno‐linguistic group was included as the African reference population in the first phase of the project (International HapMap Consortium, 2003, 2005). The 1000 Genomes Project (1000 Genomes Project Consortium et al., 2010, 2012, 2015, The 1000 Genomes Project Consortium 2015) included two Nigerian ethno‐linguistic groups: the Yoruba and the Esan. These two projects focused on population genetics characteristics such as genetic and haplotype diversity, admixture, and signatures of selection. A recent initiative that is focused on genomics in relation to health and disease is the Human Heredity and Health in Africa (H3Africa) Project. Jointly funded by the National Institutes of Health (NIH) and the Wellcome Trust, H3Africa has invested in several major grants to African investigators for genomics research, capacity building, and improving infrastructure for genome research in Africa (H3Africa Consortium, 2014). Nigerian sites are participating in several of these projects, including studies of stroke, chronic kidney disease, febrile illness, cervical cancer, glaucoma, and ELSI (bioethics) among others. Several H3Africa bioinformatics nodes are also in Nigeria. These projects are currently contributing to the development of genomics/genetics facilities (genotyping, sequencing, bioinformatics), building manpower and studying these disorders for the first time in Nigeria. More importantly, the manpower and infrastructure developed in the course of these projects would prove important to the country for developing a critical mass for medical genetics and genomic medicine.

## 8 FUTURE PROSPECTS

The status of medical genetics and genomic medicine in Nigeria is still far from meeting the needs of the population. However, the situation is rapidly changing due to recent funding opportunities for new research projects, infrastructural development, and training of the needed manpower which are creating a foundation for the development of medical genetics and genomic medicine. National policies and programs are needed to provide better screening and detection of genetic disorders, coordinate clinical management, facilitate training of medical genetics/genomics professionals, and stimulate research into genetic disorders that disproportionately impact Nigerians.

## CONFLICT OF INTEREST

The authors declare no conflict of interest.
